# Sex-Dependent Synergism of an Edible THC: CBD Formulation in Reducing Anxiety and Depressive-like Symptoms Following Chronic Stress

**DOI:** 10.2174/1570159X21666230912101441

**Published:** 2023-09-12

**Authors:** Enzo Pérez-Valenzuela, Roger Hudson, Taygun Uzuneser, Marta De Felice, Hanna Szkudlarek, Walter Rushlow, Steven R. Laviolette

**Affiliations:** 1 Addiction Research Group, Schulich School of Medicine & Dentistry, University of Western Ontario, Canada;; 2 Department of Anatomy & Cell Biology, Schulich School of Medicine & Dentistry, University of Western Ontario, Canada;; 3 Department of Psychiatry, Schulich School of Medicine & Dentistry, University of Western Ontario, Canada;; 4 Lawson Health Research Institute, Schulich School of Medicine & Dentistry, University of Western Ontario, Canada

**Keywords:** Cannabis, THC, CBD, anxiety, depression, prefrontal cortex, nucleus accumbens, chronic stress

## Abstract

Cannabis has shown therapeutic potential in mood and anxiety-related pathologies. However, the two primary constituents of cannabis, cannabidiol (CBD) and Δ-9-tetrahydrocannabinol (THC) produce distinct effects on molecular pathways in neural circuits associated with affective disorders. Moreover, it has been proposed that the combination of THC: and CBD may have unique synergistic properties. In the present study, the effects of a 1:100 THC: CBD ratio edible formulation were tested in behavioural, neuronal and molecular assays for anxiety and depressive-like endophenotypes. Adult male and female Sprague-Dawley rats were stressed for 14 days. Then, for three weeks, open field, elevated plus maze, light/dark box, social interaction, sucrose preference, and the forced swim test were performed 90 minutes after acute consumption of CBD (30 mg/kg), THC (0.3 mg/kg), or 1:100 combination of THC:CBD. After behavioural tests, *in vivo*, neuronal electrophysiological analyses were performed in the ventral tegmental area and prefrontal cortex (PFC). Furthermore, western-blot experiments examined the expression of biomarkers associated with mood and anxiety disorders, including protein kinase B (Akt), glycogen synthase kinase-3 (GSK-3), BDNF, mTOR, D1, and D2 receptor in nucleus accumbens (NAc) and PFC.Edible THC:CBD produces significant anxiolytic and antidepressant effects only in stressed male rats. In most cases, the combination of THC and CBD had stronger effects than either phytochemical alone. These synergistic effects are associated with alterations in Akt/GSK3 and D2-R expression in NAc and BDNF expression in PFC. Furthermore, THC:CBD reverses chronic stress-induced alterations in PFC neuronal activity. These findings demonstrate a novel synergistic potential for THC:CBD edible formulations in stress-related pathologies.

## 1 INTRODUCTION

Individuals with mood and anxiety disorders report greater levels of cannabis consumption, a trend that has exponentially increased over the past decades [1, 2]. The impacts of cannabis exposure on mood/anxiety-related symptoms are poorly understood; however, some clinical studies report that cannabis use may be correlated with adverse outcomes in depression [3] and accelerate the development of mood/anxiety-related symptoms [4, 5]. Nevertheless, clinical and pre-clinical research has demonstrated therapeutic potential for cannabis in treating mood/anxiety-related symptoms [6-8]. For example, pre-clinical evidence has shown a dose-dependent antidepressant-like effect of CBD in rodent models of stress-coping [9-12], learned helplessness [13] and anhedonia [14, 15]. Conversely, THC produces a U-shaped dose-response antidepressant-like effect in the forced swimming test (FST) [9, 16, 17]. Regarding anxiety-related effects, while CBD possesses primarily anxiolytic effects [18, 19], THC produces different effects depending on the dose and the species tested [19-22]. Thus, there is an urgent need for a more precise characterization of how specific cannabis components (or their combinations) may improve or potentially worsen mood and anxiety-related symptoms.

Exposure to chronic stress is associated with a higher risk of developing depressive and anxiety-related symptoms [23-25] and increased anhedonia [25-29]. Similar affective and anxiety-like symptoms are observed in pre-clinical rodent models following chronic stress exposure [28], including decreased reward sensitivity, disrupted sleep patterns and decreased locomotor activity, all core endophenotypes of depressive disorders [28].

Neural regions involved in the pathogenesis of mood and anxiety symptoms, such as the prefrontal cortex (PFC) and nucleus accumbens (NAc), are functionally impaired after chronic stress exposure [29-31]. Anatomical studies have reported diminished volume and atrophy of dendritic PFC structures following chronic stress [32]. In addition, chronic stress reduces the spontaneous neuronal activity of cortical pyramidal cells and DAergic neurons in the ventral tegmental area (VTA) [33, 34]. Furthermore, molecular signaling markers associated with depressive and anxiety-like behaviours such as the protein kinase B (Akt), glycogen-synthase-kinase-3 (GSK-3), mammalian target of rapamycin (mTOR), brain-derived neurotrophic factor (BDNF), dopamine (DA) D1, and D2 receptor subtypes are dysregulated following stress exposure [35-40].

Previous evidence has reported sex-dependent differences in the therapeutic effects of antidepressants [41]. In addition, preclinical studies have shown that females are more sensitive to treatments with tricyclic antidepressants [42], selective serotonin reuptake inhibitors [43, 44] and noradrenaline reuptake inhibitors [45]. Similarly, sex-dependent differences have been reported in the pharmacological effects of cannabinoids such as THC and CBD. For example, the analgesic effects of THC are more potent in females *vs*. male rodents [46], but CBD has been reported to be ineffective for depressive-like behaviours in females [47]. However, it is important to determine how the combined effects of THC and CBD might differentially impact affective processing relative to either cannabinoid in isolation and, more importantly, how such phenomena might differ between males *vs*. females.

The present study used an integrative combination of pre-clinical behavioural tests, neuronal electrophysiology and molecular biomarker expression assays to characterize the effects of a specific 1:100 THC: CBD ratio edible formulation in anxiety and depressive-like phenotypes in male and female rats. Our findings demonstrated that while the THC: CBD formulation displayed synergistic anxiolytic and anti-depressive-like effects in males, females showed less responsiveness to CBD, THC or their combination in the majority of behavioural tests and remarkable differences in neuronal and molecular mood and anxiety-related biomarkers following cannabinoid exposure.

## 2 MATERIALS AND METHODS

### 2.1 Animals

Male and female Sprague Dawley rats (300-350 gr) were obtained from Charles River Laboratories (Quebec). All rats were single-housed and kept at room temperature between 22-24℃ on a 12 h light/dark cycle (lights on at 7:00 EST) with access to food and water *ad libitum*. Rats were handled for one week before starting experiments. After handling sessions, rats were habituated with 1 gr of Nutella^®^ in a metal dish (6.7 cm diameter) for 60 min in the home cage. All experimental protocols adhered to institutional and governmental Animal Care Protocols and regulations.

### 2.2 Chronic Unpredictable Stress Protocol

The Chronic Unpredictable Stress (CUS) protocol was adapted from previous studies [48-50], which involved 14 consecutive days of unpredictable stress events, including supra-threshold foot shocks, overnight food and water deprivation, wet bedding, tilting of home cages at a 45° angle, and sudden light deprivation. Following the 14-day protocol, food, water, and housing conditions returned to previous controlled conditions, and animal behavioural testing began one day after CUS exposure (Table **1**).

### 2.3 Drugs

Cannabis-derived CBD and THC isolates were supplied by Canopy Health Innovations Inc. (Ontario, Canada). All studies used a dose of 30 mg/kg of CBD, 0.3 mg/kg of THC or a combination of both doses. These doses were based on previous reports demonstrating antidepressant-like effects of oral consumption of CBD and a pharmacological potentiation of THC with a ratio 1:100 THC:CBD [51, 52].

### 2.4 Behavioural Tests

#### 2.4.1 Open Field

Rats were placed in an automated open field activity chamber with dimensions of 43.38 L x 43.38 W x 30.28 H cm (Med Associates) for 15 min. The total distance covered was recorded and analyzed offline.

#### 2.4.2 Elevated Plus Maze

The rats were tested in the elevated plus-maze (EPM) to determine the anxiolytic properties of THC:CBD edible formulation. The protocol has been described previously [53]. The EPM test apparatus consisted of a center platform (10 x 10 cm) elevated 50 cm off the floor, with four arms (50 x 10 cm) at 90° to each other. Two parallel arms had walls (30 cm tall) enclosing the platform. The rat was placed in the center of the maze facing the open arm and left in the maze for 5 minutes. The percentage of time on the open arm was measured. An entry was defined when the rat had all four paws in an arm of the maze. After each test, the maze was cleaned with 50% ethanol to avoid olfactory cue bias.

#### 2.4.3 Light-dark Box Test

The light-dark box (L/D Box) test is based upon a rat’s natural aversion to bright environments and attributes greater time spent in an illuminated environment as reflecting lower anxiety levels. The test was performed as previously described [54]. The test apparatus consisted of two separated 50 x 25 x 37 cm compartments connected by a 10 x 10 cm opening. One compartment was black and covered with a black lid (the dark box). In contrast, the other compartment was white with an open top and brightly illuminated by a lamp 120 cm above the apparatus floor, providing 1500 lux at floor level. At the start of the experiment, a rat was placed in the center of the lighted box with its head facing the wall opposite the door and was allowed to freely explore both compartments for a period of 8 min. A zone entry was considered to have begun when the rat placed all four paws in that zone. Experiments were videotaped and analyzed by an experimenter blind to treatment conditions. The behaviour analyzed was the latency time between leaving the dark box and entering the light box (latency to the second transition).

#### 2.4.4 Social Motivation and Recognition Tests

Rats were tested using a social interaction procedure as described previously [55]. This task evaluates two aspects of social behaviour: (1) social motivation and (2) social recognition memory. The test was carried out in a three-chambered apparatus composed of transparent Plexiglas and a floor of black Plexiglas. The base is 120 L x 60 W cm, and each chamber is 40 L x 60 W cm and 35 H cm. Rats were habituated to the three-chambered apparatus for 13 min, 24 h before testing. At the start of the experiment, the rat was placed in a three-chambered apparatus and allowed to explore for 5 minutes. Then, an unfamiliar male rat was placed in one of the chambers in a wire cage, and the testing rat explored the apparatus for 8 minutes. During this phase, the testing rat explores the chamber containing the novel rat *vs*. the empty cage. In normal conditions, the healthy rats will spend more time in the chamber with the novel rat, showing a natural preference to socialize. At the end of this phase, a second unfamiliar rat is placed in the previously empty chamber in an 8-minute session to evaluate social memory. Control rats spend more time with the novel rat in this situation, demonstrating social novelty preference. The locations of the unfamiliar rats were counterbalanced between trials. After each test, chambers and cages were cleaned with 50% ethanol to avoid olfactory cue bias. Exploration times were recorded and used to calculate a social motivation or cognition index [time spent with a stranger (or novel stranger)/ total time exploring both rats] *100.

#### 2.4.5 Sucrose Preference

The sucrose preference test is widely used in rodents to determine changes in anhedonia states. Before conducting the tests, rats were provided 48 hours of *ad-libitum* access to a 2% sucrose solution in two bottles suspended in their home cages, without access to regular water, for acclimation to the liquid-sucrose solution. Following the acclimation phase, the bottles of sucrose were removed, and rats were deprived of fluids for 16 hours before testing. During the test phase, rats were given a choice between two bottles, one containing water and another containing 2% sucrose solution, for 1 hour [56]. The sucrose preference was calculated using the following equation:


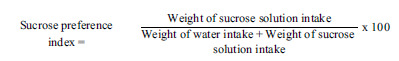


#### 2.4.6 Forced Swim Test

The FST measures acute coping behaviours reflecting anhedonia-like behaviours [57]. The rats were introduced into a cylindrical container measuring 70 × 19 cm, which was filled to three-quarters of its capacity with water at a temperature of 25ºC, and they were observed for a duration of 5 minutes. Water was replaced for each trial. The immobility times were recorded with video monitoring during the test and were analyzed individually offline.

### 2.5 Electrophysiological Recordings

One week after behavioural testing, extracellular single-unit electrophysiological recordings were simultaneously performed under urethane anesthesia (1.4 g/kg; i.p. Sigma-Aldrich) in the mPFC and the VTA, as previously described [58, 59]. Recordings were performed using glass microelectrodes (average impedance of 6-15 MΩ) filled with 2 M sodium acetate solution containing 2% pontamine sky blue (Sigma-Aldrich). Six vertical tracks separated by 200 μm were sampled at the following coordinates: for mPFC AP + 2.8 to + 3.2, ML 0.8 to 1.0 from bregma, and -2.5 to -4.0 DV from the dural surface; for VTA AP -5.0 to -5.4, ML 0.8 to 1.0 from bregma, and -6.5 to -9.0 DV from the dural surface. Extracellular signals were amplified (×5000) using a MultiClamp 700B amplifier (Molecular Devices), digitized at 25 kHz and recorded on the computer using a Digidata 1440A and pClamp software (Molecular Devices). The wideband signal of recordings was filtered to obtain single-unit recordings (band pass between 0.3 and 3 kHz). VTA DA neurons were identified according to the following well-established criteria for extracellular DA neuron identification [60, 61]: (1) action potential width > 2.2 ms, (2) spontaneous firing rate 1-10 Hz, (3) a triphasic waveform consisting of a notch on the rising phase followed by a delayed after potential, and (4) a single irregular or burst firing pattern. A burst firing pattern is defined as two consecutive spikes with an interspike interval of 80 ms. Each dopamine neuron was recorded for 5 min, and the average firing rate and the percentage of spikes that occurred in bursts were determined. Putative mPFC pyramidal cells were identified based on previously established criteria: (1) firing frequency < 10 Hz, (2) waveform shape, and (3) action potential duration > 2.5 ms. Cells were classified as burst-firing cells exhibiting three consecutive spikes with inter-spike intervals < 45 ms [54]. After an individual neuron was isolated, its spontaneous activity was recorded for 5 min. Two activity parameters were sampled, the basal firing rate and the percentage of spikes per bursting. Individual recording sessions were performed at different electrode locations throughout PFC. At the end of a neuronal recording session, electrode placements were marked using electrophoretic ejection of Pontamine Sky Blue dye from the tip of the electrode (20 μA constant negative current, 15 min) and histologically verified for correct placement using light microscopy.

### 2.6 Histological Analyses

After electrophysiology experiments, rats were decapitated under deep anesthesia (urethane 1.5 g/kg). Then, the brains were extracted, washed with NaCl 0.9% and stored in 4% paraformaldehyde. At least two days before slicing, brains were cryoprotected using a solution of 30% sucrose. Brains were frozen and sliced coronally into 50 μm sections to assess electrode recording sites. Slices were stained with Cresyl violet, and electrode recording locations were visualized under light microscopy and verified using the atlas of Paxinos & Watson (2007). Rats found with misplaced electrode recording placements were excluded from further analysis.

### 2.7 Protein Expression Analyses

Western blotting was performed as described previously [55, 56]. One week after the final behavioural experiments, rats were treated with a final exposure to THC:CBD or vehicle edibles. After 90 minutes, rats received an overdose of sodium pentobarbital (240 mg/kg, i.p.; Euthanyl), and brains were removed and flash frozen. Bilateral micro punch samples of the NAc and PFC were extracted for protein isolation. The Western blotting procedure used 12.5 µg of collected tissue per blot described previously [56]. Primary antibody dilutions were as follows: α-tubulin (1:10,000; Sigma-Aldrich), phosphorylated (p) GSK-3 α/β ser21/9 (1:1000; Cell Signaling Technology), total (t) GSK-3α/β ser21/9 (1: 250; Santa Cruz Biotechnology), p Akt (1:750; Cell Signaling Technology), t Akt (1: 1000; Cell Signaling Technology), D1 receptor (D1-R) (1: 1000; Millipore Sigma), D2 receptor (D2-R) (1: 500, Millipore Sigma), BDNF (1:1000, Abcam), p mTOR (1:1000, Cell Signaling Technology), and t mTOR (1:1000, Cell Signaling Technology). Species-appropriate fluorophore-conjugated secondary antibodies (IRDye 680RD and IRDye 800CW, LI-COR) were used at a dilution of 1:10,000. Membranes were scanned using LI-COR Odyssey Infrared Imaging System, and densitometry measurements were obtained using Image Studio analysis software. Target protein bands were normalized to the intensity of the α-tubulin control.

### 2.8 Statistical Analysis

Three-way (see supplementary data, Tables **S6**, **S7**, **S8**, **S9**) and two-way ANOVAs (See Results section) were performed for behavioural, protein expression analyses and electrophysiological recording experiments. Because the primary aim of the present study was to determine if the specific cannabinoid treatments had any impact on either male or female subjects rather than baseline differences between male *vs*. female, two-way ANOVA was used to show and discuss the results. Post hoc analyses were carried out using Holm-Sidak tests when any of the variables had a significant main effect, or the interaction effect was significant. Statistical analyses were performed using Prism GraphPad Software. All data are reported as mean ± standard error of the mean (SEM). The significance level was established at *p* < 0.05.

## 3 EXPERIMENTAL DESIGN

### 3.1 Drug Administration

CBD, THC or their combination were mixed into a chocolate hazelnut spread (Nutella^®^). The total bolus of Nutella used per serving was 6 g/kg of body weight, resulting in an edible concentration of 5mg CBD and 0.05 mg of THC per each gram of Nutella. Nutella is highly appetitive for rats; thus, food restriction was not required before the presentation of the edible samples. All edibles were prepared on the same day of the experiment. Edibles were placed in the home cage 90 minutes before each behavioural experiment and verification of consumption was performed by observation of the empty serving tray. Rats that did not voluntarily consume the edible preparation were excluded from the experiments. See details of the experimental procedure in Fig. (**1**).

## 4 RESULTS

### 4.1 Effects of Edible THC:CBD on Anxiety and Depressive-like Behavior in Chronically Stressed Male Rats

**Fig. (2):**
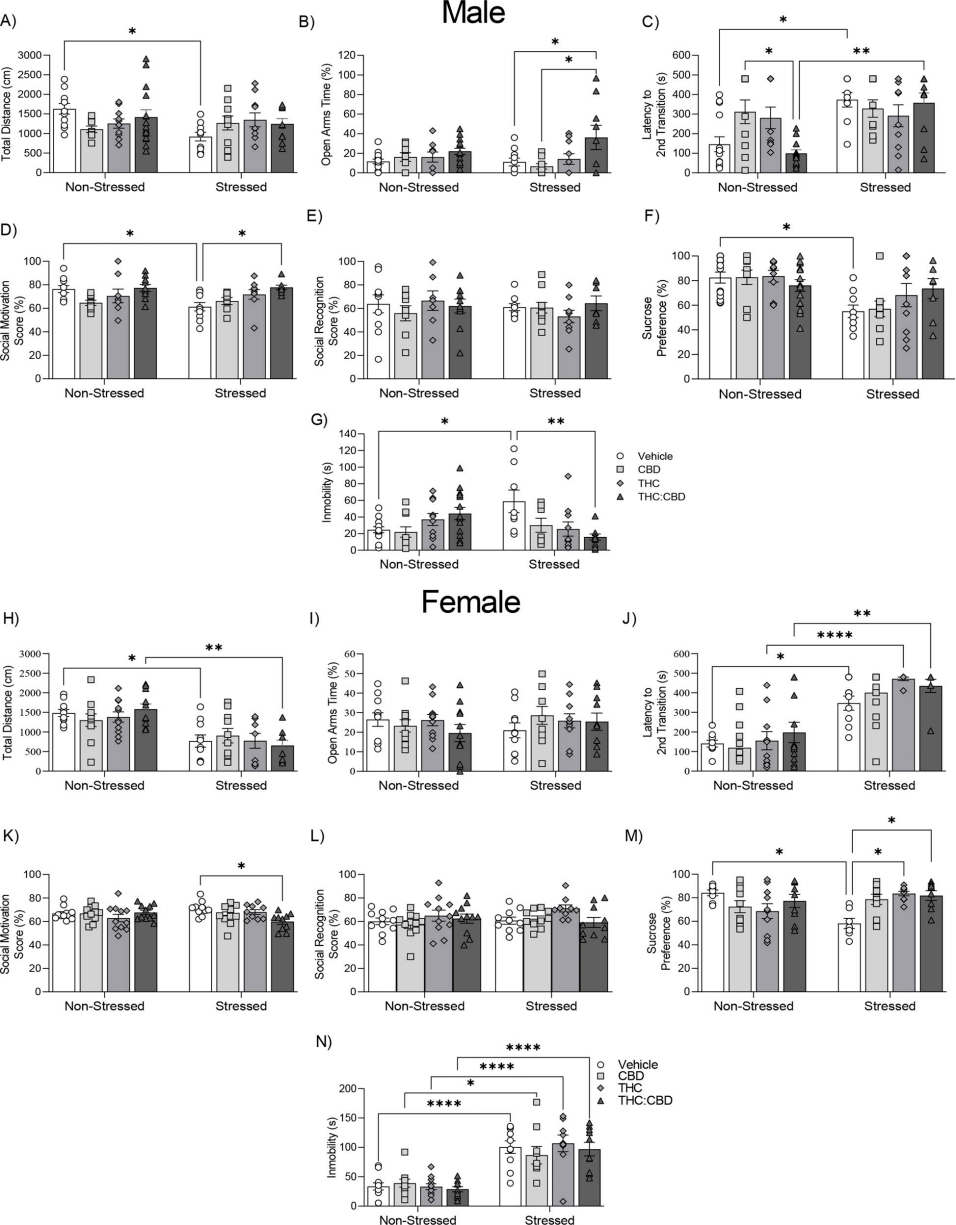
Effects of THC:CBD edible formulation on anxiety and depressive-like behaviours of non-stressed *vs.* stressed males (**A**-**G**) and female (**H**-**N**) rats. (**A**, **H**) Horizontal locomotor activity on the open field. (**B**, **I**) Open arm times. (**C**, **J**) latency time spent transitioning from the dark chamber to the lighted chamber (second transition). (**D**, **K**) Social motivation score. (**E**, **L**) Social memory. (**F**, **M**) Sucrose preference. (**G**, **N**) Time of immobility of 5 min. Data bars correspond to mean ± SEM, two-way ANOVA, Holm-Sidak post-hoc unpaired; *p < 0.05; **p < 0.01; ***p < 0.001; ****p < 0.0001.

For spontaneous locomotion tests, two-way ANOVA revealed a significant interaction between treatment x stress condition in spontaneous locomotor activity (Fig. **2A**, F_3,76_ = 3.097; *p* = 0.032). *Post-hoc* analyses revealed that chronic stress exposure decreased spontaneous locomotor activity only in the VEH control group, relative to non-stressed controls (VEH-non stressed *vs*. VEH-stressed: *p* = 0.038).

The potential anxiolytic-like effects of THC:CBD were explored using the EPM, L/D Box and social interaction tests comparing non-stressed *vs*. chronically stressed male rats. For EPM, two-way ANOVA showed a significant main effect of treatment on the percentage of open-arm times (Fig. **2B**, F_3,76_ = 5.432; *p* = 0.002). *Post-hoc* analysis showed that only the THC:CBD combination group displayed significantly increased open arm time in chronically stressed male rats *vs.* vehicle controls (VEH-stressed *vs.* THC:CBD-stressed: *p* = 0.045) and CBD groups (CBD-stressed *vs.* THC:CBD-stressed: *p* = 0.011), suggesting a synergistic effect of the THC:CBD combination *vs.* THC and CBD alone.

For the L/D box test, two-way ANOVA showed a significant effect of stress in the latency to the second transition (Fig. **2C**, F_1,77_ = 15.62; *p* = 0.0002). In addition, we observed a significant interaction between stress x treatment (F_3,77_ = 4.19; *p* = 0.008). *Post-hoc* analysis revealed an anxiogenic-like effect of chronic stress in the vehicle and THC:CBD groups (VEH-non stressed *vs.* VEH-stressed: *p* = 0.0145; THC:CBD-non stressed *vs.* THC:CBD-stressed: *p* = 0.003). Interestingly, the THC:CBD combination significantly decreased the latency to the second transition in non-stressed male rats compared to CBD alone (CBD-non stressed *vs.* THC:CBD-non stressed: *p* = 0.03).

For the social interaction test, two-way ANOVA revealed a significant main effect of treatment (Fig. **2D**, F_3,62_ = 4.083; *p* = 0.01) on social motivation scores. In addition, we observed a significant interaction between stress x treatment (F_3,62_ = 2.87; *p* = 0.044). *Post-hoc* analyses revealed that the THC:CBD combination reversed the decrease in social motivation scores induced by chronic stress exposure (VEH-non stressed *vs.* VEH-stressed *p* = 0.049; VEH-stressed *vs.* THC:CBD-stressed *p* = 0.036), while neither CBD nor THC alone reversed these effects (VEH-stressed *vs.* CBD-stressed *p* = 0.98; VEH-stressed *vs.* THC-stressed *p* = 0.5). In the social memory test, neither treatment (Fig. **2E**, F_3,62_ = 0.288; *p* = 0.834) nor stress (F_1,62_ = 0.281; *p* = 0.598) showed any significant effects. Thus, chronic stress exposure significantly decreased social motivation, and this effect was selectively reversed by THC:CBD edible treatment, relative to either cannabinoid in isolation.

The sucrose preference test is widely used in rodents to analyse changes in anhedonia based on the rodent’s natural preference to consume sweet solutions over regular water. Two-way ANOVA revealed a significant main effect of stress exposure (Fig. **2F**, F_1,74_ = 16.18; *p* = 0.0001). *Post-hoc* analyses revealed that chronic stress decreased sucrose preference in the vehicle group (VEH-non-stressed *vs.* VEH-stressed: *p* = 0.049).

In FST, immobility time is interpreted as a passive coping strategy for an inescapable situation, modelling anhedonia-like behaviours [57]. Two-way ANOVA revealed a significant treatment x stress interaction on immobility times (Fig. **2G**, F_3,74_ = 6.749; *p* = 0.0004). *Post-hoc* analysis revealed that chronic stress increased immobility times in the vehicle group (VEH-non-stressed *vs.* VEH-stressed *p* = 0.049). Furthermore, edible THC:CBD treatment reversed the increase induced by chronic stress (VEH-stressed *vs.* THC:CBD-stressed *p* = 0.008), while CBD alone and THC did not show significant effects (VEH-stressed *vs.* CBD-stressed *p* = 0.395; VEH-stressed *vs.* THC-stressed *p* = 0.98).

### 4.2 Effects of Edible THC:CBD on Anxiety and Depressive-like Behavior in Chronically Stressed Female Rats

For spontaneous locomotion tests, two-way ANOVA revealed a significant main effect of stress in spontaneous locomotor activity (Fig. **2H**, F_1,73_ = 38.77; *p* = 0.0001). *Post-hoc* analyses revealed that chronic stress exposure decreased spontaneous locomotor activity in vehicles (VEH-non stressed *vs.* VEH-stressed: *p* = 0.029) and the THC:CBD group (THC: CBD-non stressed *vs.* THC:CBD-stressed: *p* = 0.0013).

For EPM, two-way ANOVA did not reveal any significant effects of treatment (F_3,75_ = 0.38; *p* = 0.766) nor stress (F_1,75_ = 0.438; *p* = 0.51) on the percentage of open arm times (Fig. **2I**).

For the L/D box test, two-way ANOVA revealed a significant effect of stress in the latency to the second transition (Fig. **2J**, F_1,66_ = 69.07; *p* = 0.0001). *Post-hoc* analysis revealed an anxiogenic-like effect of chronic stress in the vehicle, THC and THC:CBD groups (VEH-non stressed *vs.* VEH-stressed: *p* = 0.0114; THC-non stressed *vs.* THC-stressed: *p* = 0.0001; THC:CBD-non stressed *vs.* THC:CBD-stressed: *p* = 0.002).

For the social interaction test, two-way ANOVA revealed a significant treatment x stress interaction on social motivation scores (Fig. **2K**, F_3,75_ = 3.43; *p* = 0.021). *Post-hoc* analyses revealed that only the THC:CBD combination decreased social motivation in stressed female rats (VEH-stressed *vs.* THC:CBD-stressed *p* = 0.030). In the social memory test, neither treatment (F_3,75_ = 2.324; *p* = 0.08) nor stress (F_1,75_ = 0.726; *p* = 0.397) revealed any significant effects (Fig. **2L**).

For the sucrose preference test, two-way ANOVA revealed a significant treatment x stress interaction (Fig. **2M**, F_3,62_ = 6.67; *p* = 0.0006) in female rats. *Post-hoc* analyses revealed that treatment with THC and THC:CBD reversed the decrease in sucrose preference induced by chronic stress (VEH-non stressed *vs.* VEH-stressed: *p* = 0.015; VEH-stressed *vs.* THC -stressed *p* = 0.015; VEH-stressed *vs.* THC:CBD-stressed *p* = 0.040), while having no effects in non-stressed cohorts.

In the FST, two-way ANOVA revealed a significant effect of chronic stress on immobility times (Fig. **2N**, F_1,72_ = 89.19; *p* = 0.0001). *Post-hoc* analysis revealed that chronic stress increased immobility times in vehicle (VEH-non stressed *vs.* VEH-stressed *p* = 0.0001), CBD (CBD-non stressed *vs.* CBD-stressed *p* = 0.003), THC (THC-non stressed *vs.* THC-stressed *p* = 0.0001), and THC:CBD (THC:CBD-non stressed *vs.* THC:CBD-stressed *p* = 0.0002) experimental groups, however no cannabinoid treatments reversed these effects in any cohort.

### 4.3 Effects of Edible THC:CBD on Neuronal Activity States in the PFC and VTA in Male Rats

**Fig. (3):**
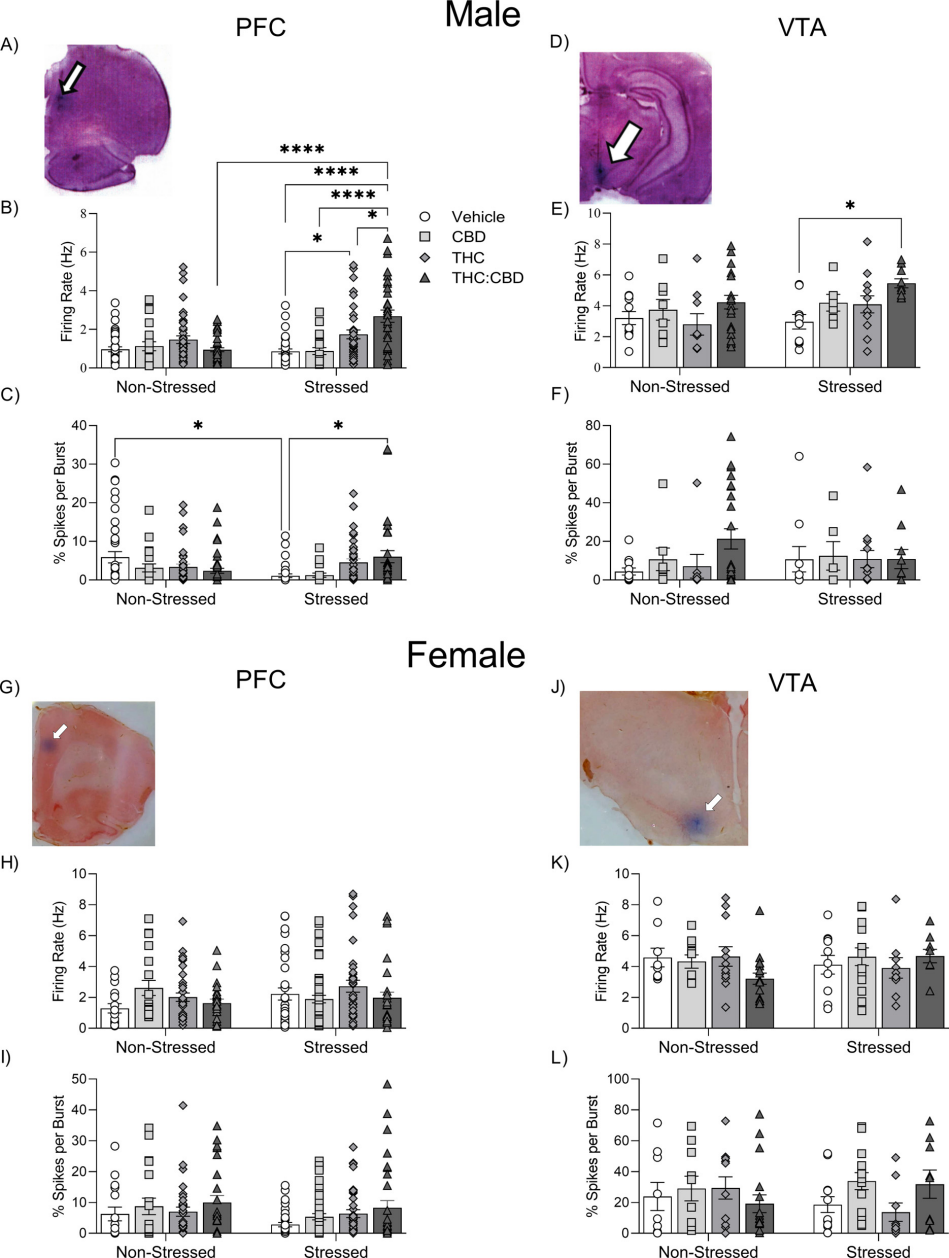
Effects of THC:CBD edible formulation on single-unit activity of pyramidal neurons recorded in PFC and DAergic neurons in the VTA of non-stressed *vs.* stressed male (**A**-**F**) and female (**G**-**L**) rats. (**A**, **G**) Representative histology example of electrode placement in PFC. (**B**, **H**) Firing rate of pyramidal neurons. (**C**, **I**) Percentage of spikes per burst of pyramidal neurons. (**D**, **J**) Representative histology example of electrode placement in VTA. (**E**, **K**) Firing rate of DAergic neurons. (**F**, **L**) Percentage of spikes per burst of DAergic neurons. Data bars correspond to mean ± SEM, two-way ANOVA, Holm-Sidak post-hoc unpaired; *p < 0.05; **p < 0.01; ***p < 0.001; **** *p* < 0.0001.

First, considering analyses of *in vivo* neuronal recordings conducted in the male PFC: Two-way ANOVA revealed a significant effect of treatment (Fig. **3A**-**L**), F_3,258_ = 10.22; *p* = 0.0001) and stress (F_1,258_ = 8.129; *p* = 0.0047) on firing rates of putative PFC pyramidal neurons. In addition, the interaction of treatment x stress was significant (Fig. **3B**) (F_3,258_ = 10.92; *p* = 0.0001). *Post-hoc* analysis revealed that stressed male rats displayed higher rates of PFC neuronal firing frequencies treated with edible THC than vehicles (VEH-stressed *vs.* THC-stressed, *p* = 0.0252). In addition, the THC:CBD-stress group showed a higher firing rate of PFC neurons compared to stressed rats treated with vehicle (VEH-stressed *vs.* THC:CBD-stressed, *p* = 0.0001), CBD (CBD-stressed *vs.* THC:CBD-stressed, *p* = 0.0001), THC (THC-stressed *vs.* THC:CBD-stressed, *p* = 0.0136) and non-stressed rats treated with the THC:CBD combo (THC:CBD-non stressed *vs.* THC:CBD-stressed, *p* = 0.0001). Thus, PFC pyramidal neurons showed increased sensitivity selectively to the THC:CBD combination following chronic stress exposure.

Considering the percentage of PFC neuronal spikes occurring in a burst pattern, two-way ANOVA revealed a significant interaction of stress x treatment (Fig. **3C**, F_3,258_ = 6.642; *p* = 0.0002). *Post-hoc* analysis revealed that chronic stress alone significantly decreased neuronal burst patterns (VEH-non stressed *vs.* VEH-stressed, *p* = 0.0231). Interestingly, only the THC:CBD treatment reversed this stress-induced effect on pyramidal neuron bursting levels (VEH-stressed *vs.* THC:CBD-stressed, *p* = 0.0272). Thus, CUS decreased the bursting activity of pyramidal neurons in the PFC. In addition, only the THC:CBD treatment reversed this effect, again suggesting a synergistic effect with the THC:CBD combination.

Following analysis of VTA DAergic neuron activity recordings, two-way ANOVA revealed a significant effect of treatment on firing rates of putative DAergic VTA neurons (Fig. **3E**, F_3,83_ = 4.567; *p* = 0.005). *Post-hoc* analysis revealed that edible THC:CBD treatment increased firing rates of DAergic neurons in the VTA only in stressed rats (VEH-stressed *vs.* THC:CBD-stressed, *p* = 0.0497). Neither CUS nor cannabinoid treatments produced any significant effects in burst firing states of putative VTA DAergic neurons (Fig. **3F**, Chronic Unpredictable Stress: F_1,83_ = 0.0065; *p* = 0.936; Treatment: F_3,83_ = 0.975; *p* = 0.409). Thus, only THC:CBD edible administration increased DAergic neuron firing rates in stressed rats, suggesting a selective synergistic effect of the THC:CBD combination.

### 4.4 Effects of Edible THC:CBD on Neuron Activity States of PFC Putative Pyramidal and VTA Putative DA in Female Rats

Considering analyses of PFC neuronal activity states in females, two-way ANOVA did not reveal significant effects of chronic stress (F_1,238_ = 1.467; *p* = 0.227) or treatment (F_3,238_ = 1.493; *p* = 0.217) on firing rates (Fig. **3H**) nor percentages of spikes per burst (Fig. **3I**, CUS: F_1,238_ = 3.473; *p* = 0.064, Treatment: F_1,238_ = 2.092; *p* = 0.102) of PFC putative pyramidal neurons.

Considering recordings performed in the VTA: two-way ANOVA did not reveal significant effects of chronic stress or treatment on DAergic neuron firing rates (Fig. **3K**, CUS: F_1,84_ = 0.131; *p* = 0.718, Treatment: F_1,84_ = 0.359; *p* = 0.783) or percentages of spikes per burst event (Fig. **3L**, CUS: F_1,84_ = 0.0297; *p* = 0.864, Treatment: F_3,84_ = 0.9282; *p* = 0.431) of recorded putative DAergic neurons in the VTA.

### 4.5 Effects of Edible THC:CBD Administration in Akt, GSK3, mTOR, BDNF, D1 and D2 Receptor Expression in NAc of Stressed Male Rats

**Fig. (4):**
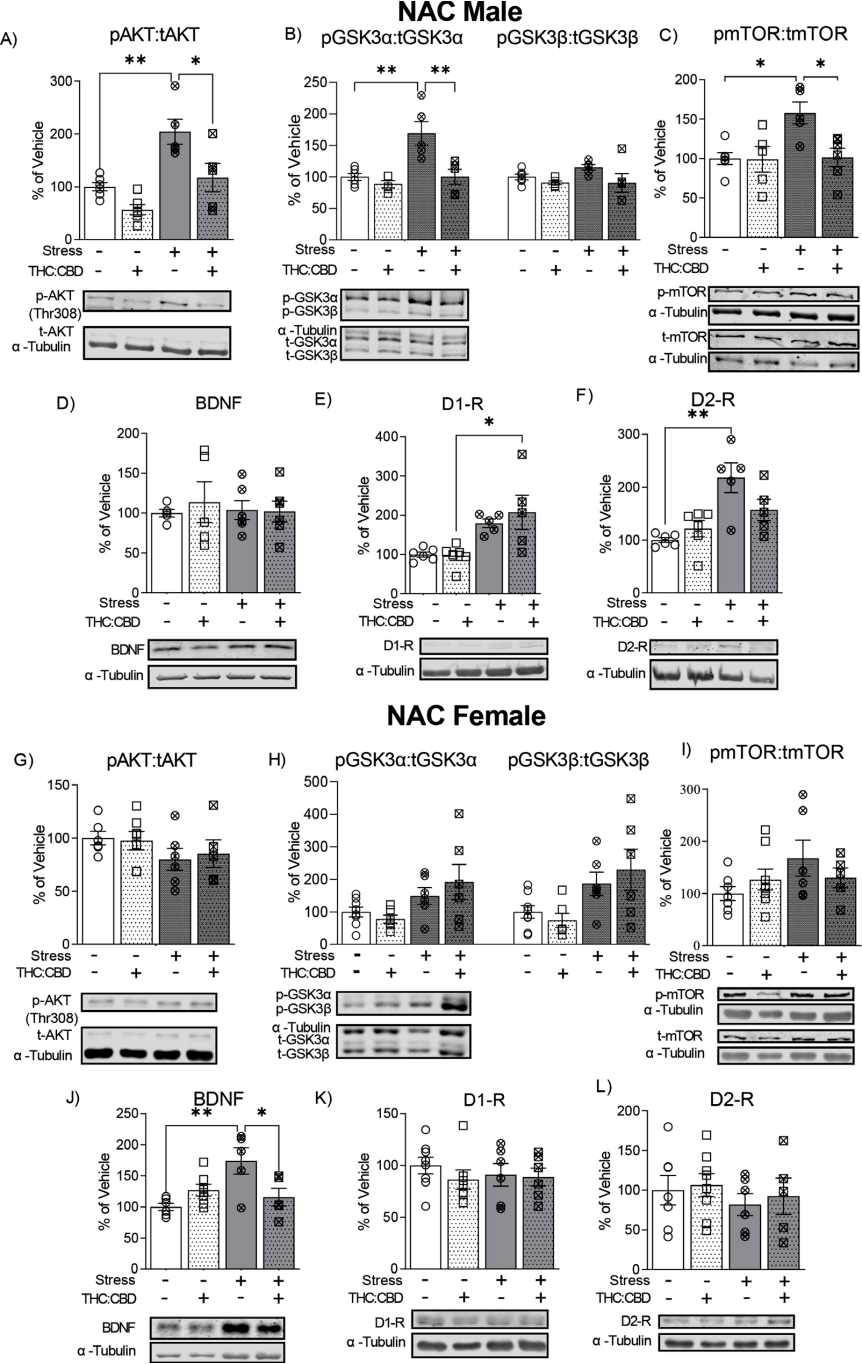
Effects of THC:CBD edible formulation on expression of Akt (**A**, **G**), GSK3 α/β (**B**, **H**), mTOR (**C**, **I**), BDNF (**D**, **J**), D1-R (**E**, **K**) and D2-R (**F**, **L**) in NAc of non-stressed and stressed male (**A**-**F**) and female (**G**-**L**) rats. Representative Western blots (Bottom) and quantification (Top) in the NAC of comparison between non-stressed and stressed cohorts. Data correspond to mean ± SEM, two-way ANOVA, Holm-Sidak post-hoc unpaired; *p < 0.05; **p < 0.01.

Two-way ANOVA showed a significant effect of chronic stress and the THC:CBD edible treatment in the ratio of p-Akt (Thr308):t-Akt (Fig. **4A**, Chronic stress: F_1,18_ = 22.03; *p* = 0.0002; Treatment: F_1,18_ = 13.79; *p* = 0.002). *Post-hoc* analysis revealed that edible THC:CBD treatment reversed the increase of p-Akt (Thr308):t-Akt expression (VEH-non stressed *vs.* VEH-stressed, *p* = 0.003; VEH-stressed *vs.* THC:CBD-stressed, *p* = 0.014) induced by CUS.

Two-way ANOVA revealed a significant effect of chronic stress and edible THC:CBD treatment in the ratio of p-GSK3α:t-GSK3α (Fig. **4B**, Chronic stress: F_1,17_ = 12.82; *p* = 0.002; Treatment: F_1,17_ = 12.74; *p* = 0.002). Furthermore, a significant stress x treatment interaction in the ratio of p-GSK3α:t-GSK3α (F_1,17_ = 6.492; *p* = 0.021) was observed. *Post hoc* analysis showed that THC:CBD treatment reversed the increase in p-GSK3α:t-GSK3α ratio (VEH-non stressed *vs.* VEH-stressed, *p* = 0.002; VEH-stressed *vs.* THC:CBD-stressed, *p* = 0.0022) induced by chronic stress. In addition, THC:CBD exposure induced a significant effect on p-GSK3β:t-GSK3β ratio expression (F_1,17_ = 4.499; *p* = 0.049; two-way ANOVA).

Two-way ANOVA showed a significant effect of chronic stress and treatment in the ratio p-mTOR:t-mTOR (Fig. **4C**, Chronic stress: F_1,18_ = 6.06, *p* = 0.024; THC:CBD F_1,18_ = 5.44, *p* = 0.031) and together with a stress x treatment interaction (F_1,18_ = 5.12; *p* = 0.036). *Post hoc* analysis revealed that edible THC:CBD treatment reversed the increase of p-mTOR:t-mTOR ratio (VEH-non stressed *vs*. VEH-stressed, *p* = 0.022; VEH-stressed *vs*. THC:CBD-stressed, *p* = 0.022) induced by CUS.

For BDNF, the treatment and CUS did not significantly effect BDNF levels in NAc (Fig. **4D**, CUS: F_1,18_ = 0.066; *p* = 0.8, Treatment: F_1,18_ = 0.151; *p* = 0.702).

Two-way ANOVA showed a significant effect of chronic stress in the expression of D1 (Fig. **4E**, F_1,18_ = 19.43; *p* = 0.0003) and D2 (Fig. **4F**, F_1,18_ = 18.16; *p* = 0.0005) receptors in NAc. In addition, a significant stress x treatment interaction in D2 receptor level expression (F_1,18_ = 5.282; *p* = 0.034) was observed. *Post-hoc* analysis revealed a significant increase of D1-R and D2-R expression induced by chronic stress (D1-R: THC:CBD-non stressed *vs.* THC:CBD-stressed, *p* = 0.012; D2-R: VEH-non stressed *vs.* VEH-stressed, *p* = 0.0012). Interestingly, the THC:CBD treatment attenuated the increase induced by chronic stress in the D2-R levels (VEH-non stressed *vs.* THC:CBD-stressed, *p* = 0.129).

### 4.6 Effects of Edible THC:CBD Administration in Akt, GSK3, BDNF, mTOR, D1 and D2 Receptor Expression in NAc of Stressed Female Rats

No significant effects of treatment or CUS were observed in the ratio p-Akt (Thr308):t-Akt in the female NAc (Fig. **4G**, CUS: F_1,20_ = 2.84; *p* = 0.1075, Treatment: F_1,20_ = 0.024; *p* = 0.877).

Two-way ANOVA revealed a significant effect of chronic stress in expression levels of p-GSK3α:t-GSK3α (Fig. **4H**, F_1,23_ = 6.42; *p* = 0.019) and p-GSK3β:t-GSK3β (F_1,23_ = 7.17; *p* = 0.013).

The treatment and CUS did not significantly affect the ratio of p-mTOR:t-mTOR levels in NAc of female rats (Fig. **4I**, CUS: F_1,22_ = 2.398; *p* = 0.136, Treatment: F_1,22_ = 0.057; *p* = 0.814).

Two-way ANOVA showed a significant effect of chronic stress (Fig. **4J**, F_1,19_ = 5.89; *p* = 0.025) in NAc. In addition, a significant stress x treatment interaction in BDNF expression (F_1,19_ = 10.82; *p* = 0.004) was observed. *Post hoc* analysis reveals that edible THC:CBD treatment reversed the increase of BDNF expression (VEH-non stressed *vs.* VEH-stressed, *p* = 0.005; VEH-stressed *vs.* THC:CBD-stressed, *p* = 0.036) induced by CUS.

The treatment and CUS did not significantly effect in the D1-R and D2-R levels in NAc of female rats (Fig. **4K**, D1-R: CUS: F_1,23_ = 0.129; *p* = 0.723, Treatment: F_1,23_ = 0.743; *p* = 0.398, Fig. **4L**, D2-R: CUS: F_1,22_ = 0.835; *p* = 0.371, Treatment: F_1,22_ = 0.233; *p* = 0.634).

### 4.7 Effects of Edible THC:CBD Administration in Akt, GSK3, mTOR, BDNF, D1 and D2 Receptor Expression in PFC of Stressed Male Rats

**Fig. (5):**
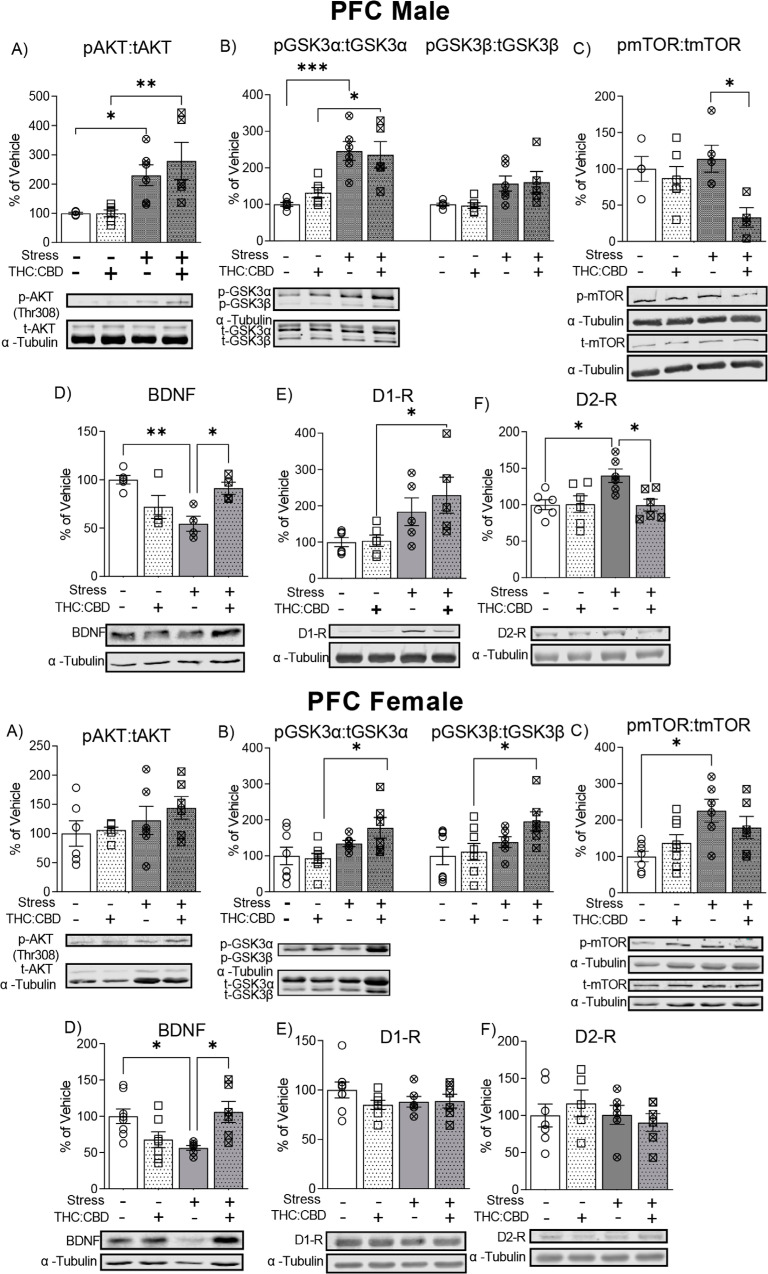
Effects of THC:CBD edible formulation on expression of Akt (**A**, **G**), GSK3 α/β (**B**, **H**), mTOR (**C**, **I**), BDNF (**D**, **J**), D1-R (**E**, **K**) and D2-R (**F**, **L**) in PFC of non-stressed and stressed male (**A**-**F**) and female (**G**-**L**) rats. Representative Western blots (Bottom) and quantification (Top) in the PFC of comparison between non-stressed and stressed cohorts. Data correspond to mean ± SEM, two-way ANOVA, Holm-Sidak post-hoc unpaired; *p < 0.05; **p < 0.01; ***p < 0.001.

Two-way ANOVA showed a significant effect of chronic stress in the ratio of p-Akt(Thr308):t-Akt (Fig. **5A**, F_1,19_ = 20.93; *p* = 0.0002). *Post-hoc* analysis revealed that CUS increased the ratio of p-Akt (Thr308): t-Akt expression in both vehicle (VEH-non stressed *vs.* VEH-stressed, *p* = 0.046) and THC:CBD treated rats (THC:CBD-non stressed *vs.* THC: CBD-stressed, *p* = 0.002; VEH-non stressed *vs.* THC:CBD-stressed, *p* = 0.01).

Two-way ANOVA showed a significant effect of chronic stress in the ratio of p-GSK3α:t-GSK3α (Fig. **5B**, F_1,19_ = 32.4; *p* = 0.0001), and the ratio of p-GSK3β:t-GSK3β (F_1,19_ = 12.21; *p* = 0.002). *Post hoc* analysis showed a higher p-GSK3α:t-GSK3α ratio in the stressed groups compared to non-stressed (VEH-non stressed *vs.* VEH-stressed, *p* = 0.0007; THC:CBD-non stressed *vs.* THC:CBD-stressed, *p* = 0.012).

Two-way ANOVA showed a significant effect of treatment in the ratio p-mTOR:t-mTOR (Fig. **5C**, F_1,15_ = 7.53, *p* = 0.015). *Post hoc* analysis showed that stressed rats treated with THC:CBD showed a lower ratio p-mTOR:t-mTOR compared to stressed vehicle group (VEH-stressed *vs.* THC:CBD-stressed, *p* = 0.044).

Two-way ANOVA showed a significant interaction between chronic stress and treatment (Fig. **5D**, F_1,13_ = 17.53; *p* = 0.001) in BDNF expression in PFC. *Post hoc* analysis reveals that THC:CBD treatment reversed the reduction of BDNF expression induced by CUS (VEH- stressed *vs.* THC:CBD-stressed, *p* = 0.03; VEH-non stressed *vs.* VEH-stressed, *p* = 0.005).

Two-way ANOVA showed a significant effect of chronic stress in the expression of D1-R (Fig. **5E**, F_1,18_ = 11.9; *p* = 0.003) and D2-R (Fig. **5F**, F_1,20_ = 4.57; *p* = 0.045) Furthermore, treatment showed a significant effect and interaction with stress in the expression of D2-R (THC:CBD: F_1,20_ = 4.79, *p* = 0.041; Interaction: F_1,20_ = 5.17, *p* = 0.034). *Post-hoc* analysis revealed a significant increase of D1-R and D2-R expression induced by chronic stress (D1-R: THC:CBD-non stressed *vs.* THC:CBD-stressed, *p* = 0.045; D2-R: VEH-non stressed *vs.* VEH-stressed, *p* = 0.030). Interestingly, THC: CBD treatment reversed stress-induced increases in D2-R expression (VEH-stressed *vs.* THC:CBD-stressed, *p* = 0.03).

### 4.8 Effects of Edible THC:CBD Administration in Akt, GSK3, mTOR, BDNF, D1 and D2 Receptor Expression in PFC of Stressed Female Rats

The treatment and CUS did not significantly effect in the ratio of p-Akt (Thr308):t-Akt levels in PFC of female rats (Fig. **5G**, CUS: F_1,21_ = 2.748; *p* = 0.1123, Treatment: F_1,21_ = 0.552; *p* = 0.466).

Two-way ANOVA showed a significant effect of chronic stress in the ratio of p-GSK3α:t-GSK3α (Fig. **5H**), (F_1,24_ = 8.36; *p* = 0.008), and the ratio of p-GSK3β:t-GSK3β (F_1,24_ = 6.78; *p* = 0.016). *Post hoc* analysis showed that p-GSK3α:t-GSK3α ratio and p-GSK3β:t-GSK3β ratio was higher in THC:CBD stressed group compared to THC:CBD non-stressed rats (p-GSK3α:t-GSK3α: THC:CBD-non stressed *vs.* THC:CBD-stressed, *p* = 0.046; p-GSK3β:t-GSK3β: THC:CBD-non stressed *vs.* THC:CBD-stressed, *p* = 0.047).

Two-way ANOVA showed a significant effect of stress in the ratio p-mTOR:t-mTOR (Fig. **5I**, F_1,23_ = 11.37, *p* = 0.003). *Post hoc* analysis showed that stress increased the ratio p-mTOR:t-mTOR (VEH-non stressed *vs.* VEH-stressed, *p* = 0.011).

In the case of BDNF in the PFC, two-way ANOVA showed a significant interaction between chronic stress and treatment (Fig. **5J**, F_1,23_ = 14.81; *p* = 0.001) in the PFC. *Post hoc* analysis reveals that THC:CBD treatment reversed the reduction of BDNF expression induced by CUS (VEH- stressed *vs.* THC:CBD-stressed, *p* = 0.03; VEH-non stressed *vs.* VEH-stressed, *p* = 0.036). Thus, chronic stress selectively downregulates BDNF levels in the PFC, and this reduction was reversed by THC:CBD edible administration.

CUS treatment had no effects on expression levels of either D1 or D2-R the female NAc (Fig. **5K**, D1-R: CUS: F_1,23_ = 0.3794; *p* = 0.544, Treatment: F_1,23_ = 1.156; *p* = 0.294, Fig. **5L**, D2-R: CUS: F_1,20_ = 0.733; *p* = 0.402, Treatment: F_1,20_ = 0.041; *p* = 0.841).

## 5 DISCUSSION

Given the highly divergent molecular, pharmacological, neuronal and behavioural effects associated with CBD *vs.* THC [62, 63], a critical translational question is whether they may produce opposing, neutral or potentially synergistic effects in combination, specifically in the context of mood and anxiety-related phenotypes. In addition, how CBD and THC may differentially impact males *vs.* females in these domains remains poorly understood. Our study provides novel evidence for a sex-dependent potentiation of the effects of CBD and THC when administered in combination, specifically in the context of depressive and anxiety-like behavioural, neuronal and molecular outcomes following chronic stress exposure.

In the present study, CUS reduced spontaneous activity and increased anxiety-like and anhedonic behaviours in both male and female rats. However, in the majority of phenotypes explored, the THC:CBD edible formulation showed ameliorative effects in males only. Further, the apparent synergism between THC and CBD was limited to male subjects as well, suggestive of critical sex differences in responsiveness to THC and CBD following chronic stress exposure. Although several studies have demonstrated the independent effects of CBD and THC in stress-related disorders [8, 9, 13, 64-68], to our knowledge, the current findings are the first evidence for increased efficacy with the combination of THC and CBD in edible formats.

In terms of observed phenotypic sex-differences, THC:CBD and THC alone treatments were able to reverse stress-induced anhedonia in females, but only in the sucrose preference test. No cannabinoid treatments produced effects in either the non-stressed or stressed cohorts in any other measure in female cohorts. Previous studies have reported sex-dependent effects for both THC and CBD on anxiety and depressive-like symptoms. For example, Salviato *et al.* (2021) reported biphasic effects of THC on anxiety-like behaviours in female rats, with females showing anxiolytic effects at lower THC (0.1 mg) doses *vs.* anxiogenic effects at higher doses (1.0 mg) [69]. While for CBD, previous studies have reported that females are resistant to the antidepressant effects of CBD [47, 70]. Although it has been hypothesized that these sex-differences may be attributed to pharmacokinetic dimorphism [71, 72], gonadal hormone influence on endocannabinoid signaling [73], or differences in cannabinoid receptor density of specific neural targets [74, 75], future studies are required to fully elucidate these sex-based differences [76] and how the mode of cannabinoid administration may contribute to these sex-selective effects.

Despite limited evidence for functional interactions between CBD and THC on anxiety and anhedonia-related phenotypes, previous evidence has suggested potential synergism between THC and CBD in other domains. For example, treatment with a 1:1 THC:CBD ratio formulation potentiated hypothermia relative to THC alone [77]. Furthermore, CBD potentiated the effects of THC in hypo-locomotor, hypothermia, antinociceptive, and drug-discrimination assays using a 1:100 THC:CBD ratio [52, 78]. This evidence is supported by pharmacokinetic studies, which have shown that CBD administration increases plasma and central THC concentrations by inhibiting THC metabolism [79, 80]. However, future studies must examine other THC:CBD ratio formulations to determine if such synergistic effects are still observable over a range of potential concentrations.

Previous reports have demonstrated that the PFC is highly sensitive to chronic stress exposure [81, 82]. These pathological effects include decreased bursting states and hypoactivity of cortical pyramidal neurons [33, 83]. In the present study, chronic stress blunted spontaneous bursting activity in PFC neurons in males and administration of the THC:CBD combination reversed this effect. Our results further demonstrated that the THC:CBD edible treatment selectively reversed stress-induced reductions in PFC bursting rates and increased average firing rates only in stressed male rats. Interestingly, previous studies have reported that excitatory optogenetic stimulation of PFC pyramidal neurons can produce long-lasting antidepressant-like effects [84, 85], suggesting that the antidepressant-like effects of the THC:CBD combination may be attributable to its ability to increase PFC neuronal activity states following chronic stress.

Previous reports have shown that acute or chronic THC can increase glutamate and/or decrease GABAergic activity in the PFC, leading to dysregulation of cortical excitatory/inhibitory balance [86, 87]. In addition, Linge *et al.* (2016) demonstrated that acute and chronic CBD treatment increased extracellular levels of serotonin and glutamate in the PFC [88]. Thus, it is possible that the combination of THC and CBD produces a complex interplay between cannabinoid and serotonergic neural substrates in the PFC that more effectively counteracts the effects of chronic stress on PFC neuronal populations than either phytochemical alone. Future studies are required to explore these possibilities.

Mesolimbic DAergic activity states are critically involved in stress and anxiety-related phenomena. Previous evidence has shown that chronic stress decreases the activity states of VTA DAergic neurons, associated with the presence of anxiety/anhedonic-like behaviours [34, 89]. However, our stress protocol did not modify the baseline activity states of VTA DA neurons in either male or female rats. Nevertheless, the THC:CBD edible treatment selectively increased DAergic neuronal firing rates in stressed males without effects in females. Previous evidence has shown that acute THC increases mesolimbic DAergic transmission, which has been attributed to an inactivation of GABAergic inputs in the VTA *via* inhibitory CB_1_R activation [90-92]. Interestingly, CBD also strongly modulates VTA DAergic neuronal activity states, but in the opposite manner. In a study by Renard *et al.* (2016), it was demonstrated that intra-NAC administration of CBD counteracts VTA DAergic neuron hyperactivity in male rats sensitized with amphetamine, *via* local modulation of the mTOR/p70S6 Kinase signaling pathway [93].

Furthermore, Norris *et al.* (2016) reported that intra-NASh CBD (in male rats) elicited a strong decrease in spontaneous VTA DAergic neuronal frequency and bursting activity through the activation of 5HT_1A_ receptor substrates. While these studies used different administration routes and only male subjects, they demonstrated that THC and CBD could differentially modulate VTA DAergic activity states *via* distinct functional mechanisms [94]. While future studies are required to explore these questions further, these distinct functional mechanisms may, in turn, serve as divergent neural pathways by which THC and CBD may synergistically modulate DAergic activity patterns in anxiogenic or anhedonic conditions.

In the present study, we observed multiple sex-dependent effects on the expression levels of several signaling pathways linked to anxiety and mood disorders. We found that CUS selectively increased the phosphorylation levels of Akt and GSK3 in both the NAc and PFC of male rats while having no observable effects in females. Consistent with these results, previous evidence has shown that CUS increases pAKT levels in the NAc and is associated with a depressive phenotype in mice [95]. Similarly, Hudson *et al.* (2020) reported increased pAkt and pGSK3 expression levels in the NAc associated with anxiety-like phenotypes induced by acute THC [56]. In addition, dysregulated Akt/GSK3 signaling in the PFC has been observed in depressed suicide victims [96, 97]. Interestingly, our findings showed that the THC:CBD combination reversed the effects of CUS on Akt/GSK3 phosphorylation levels only in NAc, having no effects in PFC. Although there is little evidence directly examining the combination of THC:CBD on Akt/GSK3 signaling, the present findings are consistent with some previous evidence. For example, Hudson *et al.* (2022) demonstrated that an acute intra-NAc infusion of THC decreased the phosphorylation states of Akt and GSK3, which was associated with anxiolytic behavioural effects and blocked by co-administration of CBD [98]. Renard *et al.* (2017) showed that acute intra-NAc CBD microinfusions decreased the phosphorylation of Akt/GSK3 in amphetamine-sensitized rats [93]. Together, this evidence further implicates the importance of the Akt/GSK3 signaling pathway in the NAc as a mechanism underlying the anxiolytic properties of THC: CBD.

BDNF dysregulation is a well-established biomarker for mood and anxiety-related disorders. Postmortem studies describe lower expression levels of PFC BDNF in suicide victims [99]. Pre-clinical research has found similar results, showing that chronic stress induces downregulation of PFC BDNF expression in concert with depressive-like phenotypes [100]. Interestingly, our results show that the THC:CBD combination selectively prevented stress-induced decreases in PFC BDNF expression levels in both male and female cohorts. Similar effects have been observed following chronic treatment with typical and atypical antidepressant medications [101, 102], suggesting that the antidepressant-like effects of THC:CBD may be mediated in part by increasing PFC BDNF levels. Previous evidence has reported that both CBD and THC administration can increase BDNF expression in PFC through separate mechanisms. Thus, while THC produces this effect *via* actions on CB1 receptors [103-105], CBD appears to modulate BDNF *via* serotoninergic signaling mechanisms [13]. Thus, one possibility is that the combination of CBD and THC may synergistically modulate central BDNF levels *via* distinct pharmacological pathways. However, considering our molecular and behavioural effects together suggests that the increased expression of PFC BDNF is not sufficient to produce significant antidepressant-like results in female rats during the FST, which may indicate a broader range of neural circuits beyond the PFC-NAc network as being impacted in females. Indeed, our results showed that CUS selectively increased BDNF expression in female NAc and not in males. This increase in stress-induced BDNF has been reported previously using a repeated social defeat paradigm [106] and is associated with depressive-like phenotypes [107]. In addition, cannabinoid treatments reversed both intra-NAc and intra-PFC BDNF expression changes in females induced by CUS, accompanied by a reversal of the sucrose preference deficit only in females. This may suggest that females may be more responsive to cannabinoid-induced changes in reward sensitivity following chronic stress but not general anhedonia or other anxiety-related phenotypes.

The overall lack of sensitivity of females to the effects of cannabinoids following chronic stress may also be related to two of our observed molecular effects in female PFC. First, estrogen is known to serve a protective function in the female PFC, specifically by preventing glutamatergic dysregulation and stress-related cognitive deficits [108]. Indeed, pre-clinical studies have shown that males in general, are more vulnerable to the effects of chronic stress [108, 109]. Thus, circulating estrogen levels might serve a neuroprotective effect in females, essentially counteracting the impacts of additional interventions through cannabinoids, as suggested by the present findings. Interestingly, we found that both BDNF and GSK-3 levels, both of which are functionally related to glutamate function in the PFC [110, 111], were significantly upregulated following THC:CBD administration. Both BDNF and GSK-3 have been reported to increase the sensitivity of the estrogen receptor signaling system [112, 113], which may have heightened the neuroprotective effects of estrogen in females, masking the potential ameliorative impacts of THC or CBD exposure, with the notable exception of the sucrose preference task, which was improved by all cannabinoid treatments in females.

Alternatively, we have reported previously that the ability of CBD to reduce anxiety-related phenotypes depends on transmission through the 5-HT_1A_ receptor subtype directly in the NAc and PFC [94, 114]. Interestingly, estradiol has been reported to cause significant de-sensitization of the 5HT_1A_ receptor [115], which could provide yet another potential mechanism for the lesser efficacy of the THC:CBD formulation in females, relative to males, with females being less sensitive to the central effects of CBD. Nevertheless, it is possible that other pre-clinical behavioural tests for anxiety or anhedonic-like symptoms may have revealed effects of THC and CBD more similar to the male cohorts. Future studies are required to explore these possibilities.

We found that the THC:CBD combination also modulated expression levels of the mTOR signaling pathway in the NAc and PFC of male rats. The mTOR pathway serves as an intracellular signal conveying downstream signaling information following the activation of α‐amino‐3‐hydroxyl‐5‐methyl‐4‐isoxazole‐propionate (AMPA) and other neurotrophic factor receptors. Interestingly, recent evidence has revealed that the anxiolytic effects of intra-NAc THC are associated with a decrease in the phosphorylation state of mTOR [98]. Similarly, intra-NAc CBD has been shown to counteract amphetamine-induced DAergic hyperactivity *via* local down-regulation of mTOR phosphorylation [93]. Our results with THC:CBD edible administration showed divergent effects such that chronic stress increased mTOR phosphorylation in the NAc, which was prevented by THC:CBD administration in male rats. In contrast, THC:CBD significantly decreased mTOR phosphorylation in the PFC in chronically stressed male cohorts. Presently, our understanding of the mechanisms of NAc-PFC mTOR phosphorylation in the context of anxiety-related phenotypes is not well understood. However, given prior evidence for convergent functional effects of CBD and THC in modulating mTOR, specifically in the NAc, these results might indicate a common mechanism for the synergistic behavioural effects of the THC:CBD combination, requiring further investigation. In addition, BDNF has been shown to activate the mTOR signaling pathway *via* eukaryotic translation initiation factor 4E-binding proteins and S6Ks protein substrates, which can, in turn, increase protein synthesis in neuronal dendrites, a potential counter-adaptation against stress effects [116]. Since we observed the effects of THC:CBD on both BDNF and mTOR pathways, one possibility is that THC:CBD may induce convergent impacts on related BDNF-mTOR signaling mechanisms as an adaptive response to chronic stress exposure.

DAergic transmission plasticity is critically involved in the regulation of mood and anxiety-related phenomena. Chronic stress exposure in males selectively increased the expression of DA D1-R levels in the NAc [39, 117] and PFC [118]. In contrast, the effects of chronic stress on DA D2-R expression have been reported to increase or decrease depending on the choice of specific stress paradigms [119, 120]. In general, given that chronic stress has been associated with blunted DAergic activity states, higher expression levels of postsynaptic DA receptors may serve as a compensatory response following decreased DAergic input from the VTA [121-123]. Additionally, our results showed that THC:CBD exposure only mitigated overexpression of the DA D2-R without impacting D1-R levels. This finding is consistent with THC:CBD related increases in firing rates observed in male VTA DA neurons after treatment. These findings may suggest a selective role for mesocorticolimbic D2-R signaling modulation and/or control of VTA DAergic activity states as factors underlying the anxiolytic effects of THC:CBD administration in chronically stressed males.

Although this study establishes several potential neurobiological mechanisms underlying THC:CBD synergism in antidepressant/anxiolytic effects in male and female rats, there are several important questions that remain to be answered. Specifically, future experiments are required to determine the potential role of gonadal hormones in the therapeutic effects of THC and CBD and how such sex-differences may divergently control male *vs.* female responsiveness to THC:CBD formulations. Available sample sizes did not permit an analysis of potential estrous cycle effects on the behavioural profiles of the female cohorts, and future studies using larger female cohort sizes may be able to explore this possibility. In addition, the characterization of a wider dose-response ratio of THC:CBD edibles is crucial to better define the optimal therapeutic range for translational clinical applications. Indeed, it is possible that different THC:CBD ratios could show divergent pharmacological effects related to depressive or anxiety phenotypes selectively and/or impact males *vs.* females differentially. In addition, it will be important to determine which THC:CBD ratio might potentially worsen affective/anxiety-related symptoms or possibly induce unwanted neuropsychiatric side-effects such as paranoia or psychotomimetic symptoms.

## CONCLUSION

In summary, while the combined effects of THC and CBD have remained elusive, our investigation identifies a wide range of sex-selective neuronal, molecular and behavioural effects that are unique to combined THC:CBD edible exposure. These findings demonstrate novel evidence for the synergistic effects of THC:CBD on anxiolytic and antidepressant-like effects in the context of chronic stress exposure. Our pre-clinical evidence suggests that edible THC: CBD treatment may be more effective in males, suggesting that combined formulations of THC:CBD may be a more effective pharmacotherapeutic avenue for stress-related pathologies. In contrast, females may be more sensitive to a broader range of cannabinoid-based treatments focusing on modulating reward sensitivity during chronic stress conditions and less sensitive to the anxiolytic effects of cannabinoids.

## Figures and Tables

**Fig. (1):**
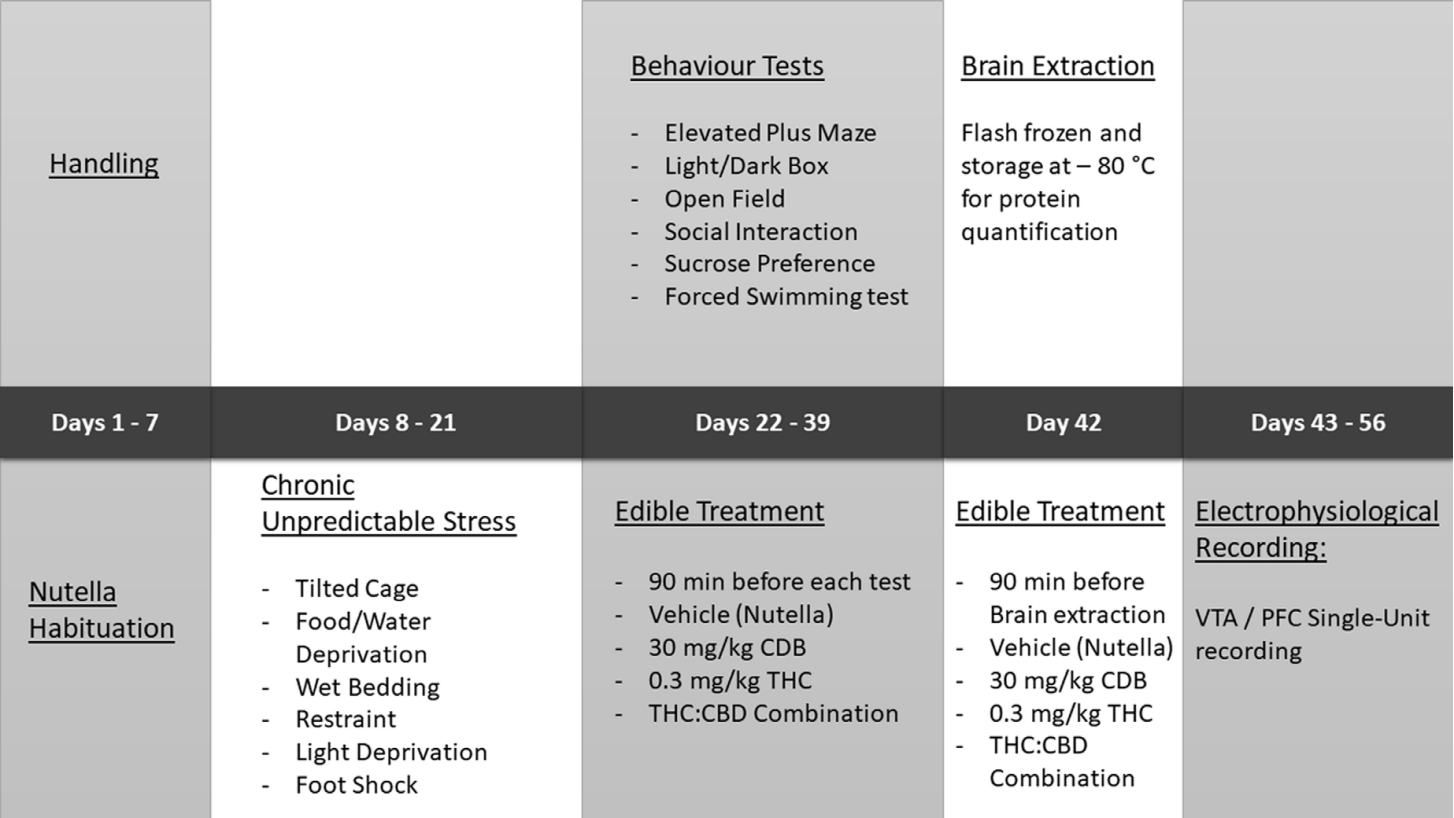
Experimental schedule.

**Table 1:** Summary of chronic unpredictable stress protocol.

**Day**	**Time**	**Stressful Event**
1	AM	2 hours - Tilted cage 45°
1	PM	Overnight - Food and water deprivation
2	AM	4 hours - Wet bedding
2	PM	1.5 hours - Restraint stress
3	AM	3 hours - Light deprivation
3	PM	2 hours - Tilted cage 45°
4	AM	10 min - 5 Foot shocks 0.6 mA
4	PM	Overnight - Food and water deprivation
5	AM	2 hours - Restraint stress
5	PM	10 min - 5 Foot shocks 0.7 mA
6	AM	4 hours - Wet bedding
7	AM	2 hours - Tilted cage 45°
7	PM	3 hours - Light deprivation
8	AM	10 min - 5 Foot shocks 0.7 mA
8	PM	4 hours - Wet bedding
9	AM	3 hours - Light deprivation
9	PM	Overnight - Food and water deprivation
10	AM	2 hours - Restraint stress
10	PM	2 hours - Tilted cage 45°
11	AM	4 hours - Wet bedding
11	PM	3 hours - Light deprivation
12	AM	2 hours - Tilted cage 45°
12	PM	10 min - 5 Foot shocks 0.8 mA
13	AM	2.5 hours - Restraint stress
13	PM	3 hours - Light deprivation
14	AM	4 hours - Wet bedding
14	PM	Overnight - Food and water deprivation

## Data Availability

Not applicable.

## LIST OF ABBREVIATIONS

Akt: Protein Kinase B
AMPA: α‐amino‐3‐hydroxyl‐5‐methyl‐4‐isoxazole‐propionate
ANOVA: Analysis of Variance
BDNF: Brain-derived Neurotrophic Factor
CBD: Cannabidiol
CUS: Chronic Unpredictable Stress
DA: Dopamine
EPM: Elevated Plus Maze
FST: Forced Swimming Test
GSK3: Glycogen Synthase Kinase-3
L/D Box: Light/Dark Box
mTOR: Mammalian Target of Rapamycin
NAc: Nucleus Accumbens
PFC: Prefrontal Cortex
THC: Δ-9-tetrahydrocannabinol
VEH: Vehicle
VTA: Ventral Tegmental Area
