# Supplementary material for: Sex-Dependent Synergism of an Edible THC: CBD Formulation in Reducing Anxiety and Depressive-like Symptoms Following Chronic Stress
Source: Curr Neuropharmacol. 2023 Sep 12;22(12):2059–78. doi: 10.2174/1570159X21666230912101441 (PMC11333796; doi:10.2174/1570159X21666230912101441)
Supplement: Supplementary file 1 [file CN-22-2059_SD1.pdf]

Supplementary Material

Sex-Dependent Synergism of an Edible THC: CBD Formulation in Reducing Anxiety and Depressive-like Symptoms Following Chronic Stress

Enzo Pérez-Valenzuela[1,2,4], Roger Hudson[1,2,4], Taygun Uzuneser[1,2,4], Marta De Felice[1,2,4], Hanna Szkudlarek[1,2,4], Walter Rushlow[1,2,3,4] and Steven R. Laviolette[1,2,3,4,*]

[1]*Addiction Research Group, Schulich School of Medicine & Dentistry, University of Western Ontario, Canada;* [2]*Department of Anatomy & Cell Biology, Schulich School of Medicine & Dentistry, University of Western Ontario, Canada;* [3]*Department of Psychiatry, Schulich School of Medicine & Dentistry, University of Western Ontario, Canada;* [4]*Lawson Health Research Institute, Schulich School of Medicine & Dentistry, University of Western Ontario, Canada*

1875-6190/24 $65.00+.00 © 2024 Bentham Science Publishers

Table S1. The number of male animals used in each behavioural experiment.

	Non-Stressed				Stressed			
	**Vehicle**	**CBD**	**THC**	**THC:CBD**	**Vehicle**	**CBD**	**THC**	**THC:CBD**
**Open Field**	11	8	10	15	10	10	10	10
**EPM**	14	8	9	15	10	9	10	9
**L/D Box**	14	10	10	12	9	10	10	10
**Social Interaction**	10	8	8	10	10	7	9	8
**Sucrose Preference**	13	9	9	15	9	9	10	8
**FST**	13	10	10	14	8	7	10	10

Table S2. The number of female animals used in each behavioural experiment.

	Non-Stressed				Stressed			
	**Vehicle**	**CBD**	**THC**	**THC:CBD**	**Vehicle**	**CBD**	**THC**	**THC:CBD**
**Open Field**	10	11	11	11	10	10	9	9
**EPM**	11	11	11	11	10	10	10	10
**L/D Box**	9	10	10	11	9	9	8	8
**Social Interaction**	11	11	11	11	10	10	9	10
**Sucrose Preference**	8	10	10	9	7	9	9	8
**FST**	11	10	11	10	10	9	9	10

Table S3. The number of recorded cells in each electrophysiological experiment.

		Non-Stressed				Stressed			
		**Vehicle**	**CBD**	**THC**	**THC:CBD**	**Vehicle**	**CBD**	**THC**	**THC:CBD**
**Male**	**PFC**	38	21	42	44	33	19	37	32
	**VTA**	12	8	8	23	10	6	13	10
**Female**	**PFC**	16	19	35	24	34	50	37	31
	**VTA**	9	10	12	17	12	14	9	9

Table S4. The number of male animals used in each molecular experiment.

		Non-Stressed		Stressed	
		**Vehicle**	**THC:CBD**	**Vehicle**	**THC:CBD**
**NAC**	**pAkt:tAkt**	6	6	5	5
	**pGSK3α:tGSK3α**	6	5	5	5
	**pGSK3β:tGSK3β**	6	5	5	5
	**pmTOR:tmTOR**	6	5	5	6
	**BDNF**	5	5	6	6
	**D1-R**	6	6	5	5
	**D2-R**	6	6	6	6
**PFC**	**pAkt:tAkt**	6	6	6	5
	**pGSK3α:tGSK3α**	6	6	6	5
	**pGSK3β:tGSK3β**	6	6	6	5
	**pmTOR:tmTOR**	4	6	5	4
	**BDNF**	5	4	4	4
	**D1-R**	6	6	5	5
	**D2-R**	6	6	6	6

Table S5. The number of female animals used in each molecular experiment.

		Non-Stressed		Stressed	
		Vehicle	THC:CBD	Vehicle	THC:CBD
NAC	pAkt:tAkt	6	7	6	5
	pGSK3α:tGSK3α	8	6	6	6
	pGSK3β:tGSK3β	8	6	6	6
	pmTOR:tmTOR	7	8	6	5
	BDNF	6	7	5	5
	D1-R	8	8	6	6
	D2-R	7	8	6	5
PFC	pAkt:tAkt	6	7	6	6
	pGSK3α:tGSK3α	8	8	6	6
	pGSK3β:tGSK3β	8	8	6	6
	pmTOR:tmTOR	7	8	6	6
	BDNF	8	7	6	6
	D1-R	8	7	6	6
	D2-R	7	5	6	6

Table S6. Three-way ANOVA (Treatment x Stress x Sex) in behavioural tests.

	Total Distance (Open Field)		Open arms time (EPM)		Second Latency (L/D Box)		Social Motivation	
	F (DFn, DFd)	P value	F (DFn, DFd)	P value	F (DFn, DFd)	P value	F (DFn, DFd)	P value
Treatment	F (3, 149) = 0.1880	P=0.9044	F (3, 152) = 2.643	P=0.0513	F (3, 143) = 0.9266	P=0.4297	F (3, 137) = 1.881	P=0.1357
Stress	F (1, 149) = 28.20	P<0.0001	F (1, 152) = 0.1628	P=0.6871	F (1, 143) = 69.44	P<0.0001	F (1, 137) = 0.976	P=0.3250
Sex	F (1, 149) = 4.765	P=0.0306	F (1, 152) = 11.32	P=0.0010	F (1, 143) = 0.2086	P=0.6486	F (1, 137) = 10.57	P=0.0014
Treatment x Stress	F (3, 149) = 3.057	P=0.0303	F (3, 152) = 1.831	P=0.1439	F (3, 143) = 2.184	P=0.0925	F (3, 137) = 1.813	P=0.1477
Treatment x Sex	F (3, 149) = 0.1686	P=0.9174	F (3, 152) = 4.601	P=0.0041	F (3, 143) = 2.078	P=0.1058	F (3, 137) = 5.523	P=0.0013
Stress x Sex	F (1, 149) = 10.69	P=0.0013	F (1, 152) = 0.03170	P=0.8589	F (1, 143) = 6.111	P=0.0146	F (1, 137) = 1.102	P=0.2958
Treatment x Stress x Sex	F (3, 149) = 1.259	P=0.2907	F (3, 152) = 1.193	P=0.3144	F (3, 143) = 3.259	P=0.0234	F (3, 137) = 4.510	P=0.0047
	Social Recognition		Sucrose Preference		Immobility Time (FST)			
	F (DFn, DFd)	P value	F (DFn, DFd)	P value	F (DFn, DFd)	P value		
Treatment	F (3, 137) = 0.6972	P=0.5553	F (3, 136) = 1.319	P=0.2709	F (3, 146) = 1.035	P=0.3792		
Stress	F (1, 137) = 0.0011	P=0.9738	F (1, 136) = 9.909	P=0.0020	F (1, 146) = 56.15	P<0.0001		
Sex	F (1, 137) = 0.3001	P=0.5847	F (1, 136) = 1.311	P=0.2543	F (1, 146) = 60.16	P<0.0001		
Treatment x Stress	F (3, 137) = 0.5522	P=0.6475	F (3, 136) = 4.990	P=0.0026	F (3, 146) = 2.367	P=0.0732		
Treatment x Sex	F (3, 137) = 1.015	P=0.3880	F (3, 136) = 0.1914	P=0.9021	F (3, 146) = 0.4771	P=0.6987		
Stress x Sex	F (1, 137) = 0.8496	P=0.3583	F (1, 136) = 9.688	P=0.0023	F (1, 146) = 53.98	P<0.0001		
Treatment x Stress x Sex	F (3, 137) = 1.217	P=0.3058	F (3, 136) = 1.908	P=0.1312	F (3, 146) = 3.524	P=0.0166		

Table S7. Three-way ANOVA (Treatment x Stress x Sex) in electrophysiological measures of PFC and VTA neurons.

	Firing Rate - PFC		% Burst - PFC		Firing Rate - VTA		% Burst - VTA	
	F (DFn, DFd)	P value	F (DFn, DFd)	P value	F (DFn, DFd)	P value	F (DFn, DFd)	P value
Treatment	F (3, 496) = 3.923	P=0.0087	F (3, 496) = 2.593	P=0.0520	F (3, 167) = 1.344	P=0.2619	F (3, 166) = 1.243	P=0.2958
Stress	F (1, 496) = 6.279	P=0.0125	F (1, 496) = 3.807	P=0.0516	F (1, 167) = 2.163	P=0.1433	F (1, 166) = 0.006	P=0.9383
Sex	F (1, 496) = 24.09	P<0.0001	F (1, 496) = 22.74	P<0.0001	F (1, 167) = 2.285	P=0.1325	F (1, 166) = 17.67	P<0.0001
Treatment x Stress	F (3, 496) = 4.360	P=0.0048	F (3, 496) = 2.899	P=0.0346	F (3, 167) = 1.749	P=0.1590	F (3, 166) = 0.347	P=0.7911
Treatment x Sex	F (3, 496) = 3.253	P=0.0215	F (3, 496) = 1.664	P=0.1739	F (3, 167) = 3.191	P=0.0251	F (3, 166) = 0.417	P=0.7414
Stress x Sex	F (1, 496) = 0.1157	P=0.7339	F (1, 496) = 1.624	P=0.2031	F (1, 167) = 0.9312	P=0.3360	F (1, 166) = 0.033	P=0.8560
Treatment x Stress x Sex	F (3, 496) = 3.494	P=0.0156	F (3, 496) = 0.950	P=0.4160	F (3, 167) = 0.8522	P=0.4673	F (3, 166) = 2.246	P=0.0849

Table S8. Three-way ANOVA (Treatment x Stress x Sex) of molecular markers in NAc.

	pAkt:tAkt		pGSK3α:tGSK3α		pGSK3β:tGSK3β		pmTOR:tmTOR	
	F (DFn, DFd)	P value	F (DFn, DFd)	P value	F (DFn, DFd)	P value	F (DFn, DFd)	P value
Treatment	F (1, 38) = 10.62	P=0.0024	F (1, 37) = 0.288	P=0.5950	F (1, 39) = 0.041	P=0.8408	F (1, 40) = 1.544	P=0.2213
Stress	F (1, 38) = 11.48	P=0.0016	F (1, 37) = 14.27	P=0.0006	F (1, 39) = 9.335	P=0.0040	F (1, 40) = 5.747	P=0.0213
Sex	F (1, 38) = 8.692	P=0.0054	F (1, 37) = 0.316	P=0.5777	F (1, 39) = 5.334	P=0.0263	F (1, 40) = 1.457	P=0.2345
Treatment x Stress	F (1, 38) = 0.827	P=0.3688	F (1, 37) = 0.044	P=0.8356	F (1, 39) = 0.4062	P=0.5276	F (1, 40) = 4.788	P=0.0346
Treatment x Sex	F (1, 38) = 11.65	P=0.0015	F (1, 37) = 3.064	P=0.0883	F (1, 39) = 0.3657	P=0.5488	F (1, 40) = 0.7104	P=0.4043
Stress x Sex	F (1, 38) = 25.45	P<0.0001	F (1, 37) = 2.201	P=0.1464	F (1, 39) = 7.321	P=0.0101	F (1, 40) = 0.0391	P=0.8443
Treatment x Stress x Sex	F (1, 38) = 1.733	P=0.1960	F (1, 37) = 2.026	P=0.1630	F (1, 39) = 1.002	P=0.3230	F (1, 40) = 0.0278	P=0.8685
	BDNF		D1-R		D2-R			
	F (DFn, DFd)	P value	F (DFn, DFd)	P value	F (DFn, DFd)	P value		
Treatment	F (1, 37) = 0.2367	P=0.6295	F (1, 41) = 0.039	P=0.8444	F (1, 40) = 0.198	P=0.6585		
Stress	F (1, 37) = 1.889	P=0.1776	F (1, 41) = 17.65	P=0.0001	F (1, 40) = 5.808	P=0.0206		
Sex	F (1, 37) = 5.886	P=0.0203	F (1, 41) = 24.81	P<0.0001	F (1, 40) = 18.37	P=0.0001		
Treatment x Stress	F (1, 37) = 6.341	P=0.0163	F (1, 41) = 0.950	P=0.3354	F (1, 40) = 2.436	P=0.1264		
Treatment x Sex	F (1, 37) = 1.172	P=0.2860	F (1, 41) = 0.853	P=0.3610	F (1, 40) = 1.246	P=0.2710		
Stress x Sex	F (1, 37) = 3.126	P=0.0853	F (1, 41) = 20.29	P<0.0001	F (1, 40) = 13.54	P=0.0007		
Treatment x Stress x Sex	F (1, 37) = 3.030	P=0.0901	F (1, 41) = 0.199	P=0.6571	F (1, 40) = 2.968	P=0.0926		

Table S9. Three-way ANOVA (Treatment x Stress x Sex) of molecular markers in PFC.

	pAkt:tAkt	pGSK3α:tGSK3α	pGSK3β:tGSK3β	pmTOR:tmTOR

	**F (DFn, DFd)**	**P value**	**F (DFn, DFd)**	**P value**	**F (DFn, DFd)**	**P value**	**F (DFn, DFd)**	**P value**
**Treatment**	F (1, 40) = 1.034	P=0.3154	F (1, 43) = 0.951	P=0.3350	F (1, 43) = 1.322	P=0.2565	F (1, 38) = 2.355	P=0.1332
**Stress**	F (1, 40) = 24.28	P<0.0001	F (1, 43) = 37.35	P<0.0001	F (1, 43) = 16.03	P=0.0002	F (1, 38) = 3.673	P=0.0628
**Sex**	F (1, 40) = 9.936	P=0.0031	F (1, 43) = 11.88	P=0.0013	F (1, 43) = 0.2618	P=0.6115	F (1, 38) = 21.50	P<0.0001
**Treatment x Stress**	F (1, 40) = 0.7455	P=0.3931	F (1, 43) = 0.015	P=0.9031	F (1, 43) = 0.7611	P=0.3878	F (1, 38) = 5.188	P=0.0284
**Treatment x Sex**	F (1, 40) = 0.0843	P=0.7731	F (1, 43) = 0.064	P=0.8009	F (1, 43) = 1.266	P=0.2668	F (1, 38) = 1.643	P=0.2077
**Stress x Sex**	F (1, 40) = 10.93	P=0.0020	F (1, 43) = 4.688	P=0.0360	F (1, 43) = 0.0013	P=0.9716	F (1, 38) = 9.724	P=0.0035
**Treatment x Stress x Sex**	F (1, 40) = 0.1979	P=0.6588	F (1, 43) = 2.359	P=0.1319	F (1, 43) = 0.4216	P=0.5196	F (1, 38) = 0.056	P=0.8140
	**BDNF**		**D1-R**		**D2-R**			
	**F (DFn, DFd)**	**P value**	**F (DFn, DFd)**	**P value**	**F (DFn, DFd)**	**P value**		
**Treatment**	F (1, 36) = 0.7454	P=0.3936	F (1, 41) = 0.379	P=0.5418	F (1, 40) = 0.9516	P=0.3352		
**Stress**	F (1, 36) = 1.125	P=0.2959	F (1, 41) = 12.78	P=0.0009	F (1, 40) = 0.1537	P=0.6971		
**Sex**	F (1, 36) = 0.1721	P=0.6807	F (1, 41) = 20.57	P<0.0001	F (1, 40) = 0.9008	P=0.3483		
**Treatment x Stress**	F (1, 36) = 24.22	P<0.0001	F (1, 41) = 1.037	P=0.3145	F (1, 40) = 3.870	P=0.0561		
**Treatment x Sex**	F (1, 36) = 0.0838	P=0.7739	F (1, 41) = 1.270	P=0.2664	F (1, 40) = 1.751	P=0.1933		
**Stress x Sex**	F (1, 36) = 0.4944	P=0.4865	F (1, 41) = 14.96	P=0.0004	F (1, 40) = 3.431	P=0.0714		
**Treatment x Stress x Sex**	F (1, 36) = 0.3339	P=0.5670	F (1, 41) = 0.211	P=0.6485	F (1, 40) = 0.1763	P=0.6768		
